# Influence of *cis* Element Arrangement on Promoter Strength in Trichoderma reesei

**DOI:** 10.1128/AEM.01742-17

**Published:** 2017-12-15

**Authors:** Daniel P. Kiesenhofer, Robert L. Mach, Astrid R. Mach-Aigner

**Affiliations:** a Research Area Biochemical Technology, Institute of Chemical, Environmental and Biological Engineering, TU Wien, Vienna, Austria; Kyoto University

**Keywords:** Trichoderma reesei, *cbh1*, *xyn1*, *cis* element, Xyr1-binding site, promoter strength, promoter inducibility, promoters, regulation of gene expression

## Abstract

Trichoderma reesei can produce up to 100 g/liter of extracellular proteins. The major and industrially relevant products are cellobiohydrolase I (CBHI) and the hemicellulase XYNI. The genes encoding both enzymes are transcriptionally activated by the regulatory protein Xyr1. The first 850 nucleotides of the *cbh1* promoter contain 14 Xyr1-binding sites (XBS), and 8 XBS are present in the *xyn1* promoter. Some of these XBS are arranged in tandem and others as inverted repeats. One such *cis* element, an inverted repeat, plays a crucial role in the inducibility of the *xyn1* promoter. We investigated the impact of the properties of such *cis* elements by shuffling them by insertion, exchange, deletion, and rearrangement of *cis* elements in both the *cbh1* and *xyn1* promoter. A promoter-reporter assay using the Aspergillus niger *goxA* gene allowed us to measure changes in the promoter strength and inducibility. Most strikingly, we found that an inverted repeat of XBS causes an important increase in *cbh1* promoter strength and allows induction by xylan or wheat straw. Furthermore, evidence is provided that the distances of *cis* elements to the transcription start site have important influence on promoter activity. Our results suggest that the arrangement and distances of *cis* elements have large impacts on the strength of the *cbh1* promoter, whereas the sheer number of XBS has only secondary importance. Ultimately, the biotechnologically important *cbh1* promoter can be improved by *cis* element rearrangement.

**IMPORTANCE** In the present study, we demonstrate that the arrangement of *cis* elements has a major impact on promoter strength and inducibility. We discovered an influence on promoter activity by the distances of *cis* elements to the transcription start site. Furthermore, we found that the configuration of *cis* elements has a greater effect on promoter strength than does the sheer number of transactivator binding sites present in the promoter. Altogether, the arrangement of *cis* elements is an important factor that should not be overlooked when enhancement of gene expression is desired.

## INTRODUCTION

Trichoderma reesei is a saprotrophic fungus that is used for industrial enzyme production. Certain strains are able to produce up to 100 g/liter of protein that is secreted into the medium (M. Ward, unpublished data). The induction signals for the expression of cellulases and hemicellulases are related to their respective substrates: cellulose and its soluble degradation products induce cellulase-encoding gene expression, and xylan and its soluble degradation products induce hemicellulase-encoding gene expression. The industrially most relevant enzyme, cellobiohydrolase I (CBHI), can make up to 60% of the secreted protein (e.g., see references 1 to 3).

**FIG 1:**
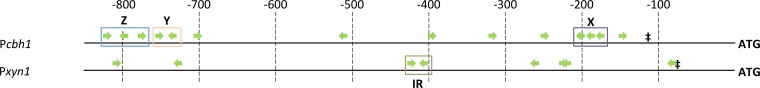
Schematic representation of the *cbh1* and *xyn1* promoters. Green arrows indicate XBS and their orientations, ‡ indicates the TATA signal, and ATG represents the start codon of the *cbh1* or *xyn1* gene. The blue box indicates the *cis* element Z (direct repeat of three XBS), the orange box indicates the *cis* element Y (an inverted repeat of XBS), the purple box indicates the *cis* element X (direct repeat of three XBS), and the brown box indicates the *cis* element IR (inverted repeat of XBS). Numbers on top indicate the distance from the ATG in base pairs.

Currently, the main research efforts focus on the transcriptional regulation of cellulase- and hemicellulase-encoding genes. Nonetheless, the impact of the arrangement of *cis* elements on promoter strength has hardly been investigated so far. Therefore, during this study, we investigated the role of *cis* elements composed of Xyr1-binding sites (XBS) in the promoters of the *cbh1* and *xyn1* genes. Xyr1 is the main and essential transactivator of *cbh1* and *xyn1* gene expression (4) and has the binding consensus sequence 5′-GGC(A/T)_3_-3′ (5). Corresponding schemes of the two promoters studied are depicted in Fig. 1.

The *xyn1* gene is positively transcriptionally regulated by Xyr1 (4). The promoter region spanning from position −1 to −850 bp contains eight XBS (Fig. 1). Gene transcription is induced by xylan and its degradation product d-xylose. Notably, d-xylose induction is concentration dependent: low concentrations at around 0.5 mM induce gene transcription, but high concentrations, e.g., 1% (wt/vol) (66.6 mM), repress *xyn1* transcription (6). On the other hand, Ace1 and Cre1 act as repressors of *xyn1* transcription (7, 8). A region between positions −321 and −538 bp in the *xyn1* promoter was identified to be necessary for the induction of the gene (6), and later, it was reported that this fragment contains all *cis* elements for the regulation of the *xyn1* gene (5). Rauscher and coworkers identified an important inverted repeat of XBS and observed that the deletion of Cre1-binding sites had no influence on the inducibility of *xyn1* (5). These results encouraged us to investigate the impact of the identified inverted repeat of XBS (located between −405 and −426 bp) in greater detail. The corresponding sequence, 5′-GGCTAAATGCGACATCTTAGCC-3′ (XBS are underlined), is termed *cis* element IR in this study.

The transcriptional activators for the *cbh1* gene are Xyr1 and Ace2 (4, 9). Cellulose and its degradation products induce gene transcription to a certain extent, while the strongest known inducer of cellulase expression is the transglycosilation product sophorose, a disaccharide formed by two β-1,2-linked glucose residues. As sophorose is a very expensive compound, the search for an equivalent replacement is still an important issue (10). Another inducing signal for *cbh1* expression is lactose, which is also mediated by Xyr1, but not by Ace2 (11, 12). The *cbh1* core promoter from position −1 to −850 bp contains 14 XBS (Fig. 1). The regulatory proteins Cre1 and Ace1 are repressors of transcription of the *cbh1* gene. Cre1 mediates carbon catabolite repression (CCR) by binding Cre1-binding sites (5′-SYGGRG-3′) (13) in the 5′ region of the *cbh1* core promoter in the presence of d-glucose. Ace1 binds DNA motifs similar to those bound by Xyr1. There are eight confirmed target sites present in the *cbh1* promoter (14). It is hypothesized that the regulation is based on competition for these sites between Xyr1 and Ace1 (7). Liu and coworkers deleted Cre1-binding sites in the *cbh1* promoter of the T. reesei Rut-C30 strain M3 and inserted the *cbh1* promoter sequence spanning from −620 to −820 bp three more times into the 5′ region (15). This approach yielded two-times-higher activity than the wild-type (WT) promoter construct using a glucuronidase reporter assay. Another approach was the exchange of Cre1-binding sites for Ace2-binding sites and binding sites for the Hap2/3/5 complex (16). This experiment was performed in the protease-deficient Rut-C30 strain RC30-8 and resulted in a 5.5-fold increase in enhanced green fluorescent protein (eGFP) expression on inducing medium by a single copy of the inserted reporter gene. The high abundance of XBS in the *cbh1* makes these sites an interesting target for research (17). In the present study, we examined three different *cis* elements composed of XBS in the *cbh1* promoter, including (i) a direct repeat of three XBS located at bp −173 of the *cbh1* promoter, 5′-TTAGCCAAGAACAATAGCCGATAAAGATAGCC-3′ (termed *cis* element X in this study), (ii) an inverted repeat of XBS located at −733 bp of the *cbh1* promoter, 5′-GGCTAAACGTACCGTAATTTGCC-3′ (termed *cis* element Y in this study), and (iii) three XBS located at −773 bp of the *cbh1* promoter, 5′-GGCTAAAAGTACATAAGTTAATGCCTAAAGAAGTCATATACCAGCGGCTAA-3′ (termed *cis* element Z in this study). The *cis* elements Y and Z are located on the same sequence stretch that was used in the study of Liu et al. (15). *cis* element X is a direct repeat of three XBS; however, it is located much closer to the TATA signal (Fig. 1).

There is only limited and rather general information about the impact of longer sequence stretches within the *cbh1* and *xyn1* core promoters, and there are no detailed investigations on the influence of *cis* elements on the inducibility and strength of these promoters. Therefore, we aimed to address the following questions during this study. (i) Is it possible to assign carbon source inducibility to certain *cis* elements and thereby modify promoter inducibility in a targeted manner by *cis* element rearrangement? (ii) What is the impact of the number of XBS on promoter inducibility and strength? (iii) Does the distance between *cis* elements and the transcription start site (TSS) have an influence on promoter activity? Certainly, the answers to these questions are important for improving the efficiency, cost-effectiveness, and productivity of target gene expression.

## RESULTS

### The *cis* element IR increases promoter strength.

**FIG 2:**
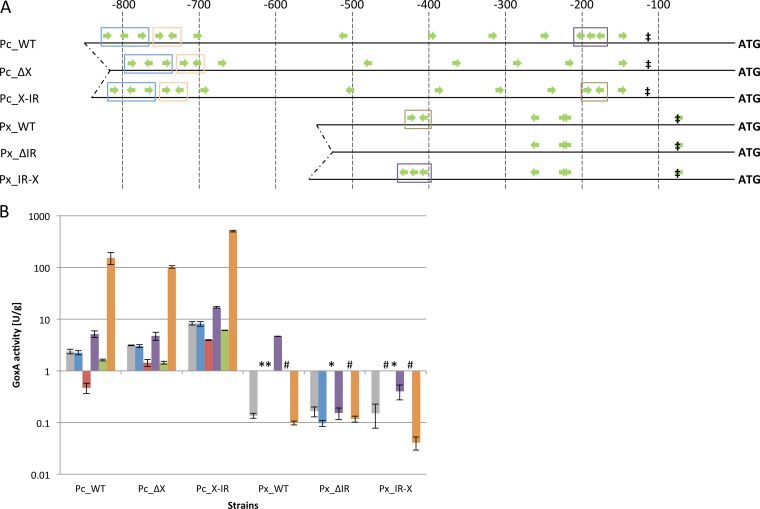
Promoter schemes and GoxA activities of recombinant T. reesei strains after a carbon source replacement experiment. (A) Green arrows indicate XBS and their orientations, ‡ indicates the TATA signal, and ATG represents the start codon of the reporter gene *goxA*. Blue boxes indicate *cis* element Z, orange boxes indicate *cis* element Y, purple boxes indicate *cis* element X, and brown boxes indicate *cis* element IR. Numbers on top indicate the distance from the ATG in base pairs. (B) Indicated strains were pregrown, and then mycelia were transferred to MA medium without any carbon source (gray bars) or with 1% glycerol (blue bars), 1% d-glucose (red bars), 0.5 mM d-xylose (purple bars), 1% d-xylose (green bars), or 1.5 mM sophorose (orange bars), incubated for 8 h, and harvested, and GoxA activities were measured in the supernatants. GoxA activity is given in relation to biomass (dry weight). Symbols indicate results below quantification level (*) and results below the detection limit (#). The mean values shown are calculated from the results of biological duplicates and technical triplicates. Error bars indicate standard deviations.

At the beginning of our studies, we were interested in whether the *cis* element X is responsible for sophorose induction in the *cbh1* promoter on the one hand and, on the other hand, whether the *cis* element IR is responsible for d-xylose induction in the *xyn1* promoter. Therefore, we constructed expression cassettes bearing the deletion of the respective *cis* element in each promoter or a motif exchange where X was exchanged for IR in the *cbh1* promoter and termed *cis* element X-IR and IR was exchanged for X in the *xyn1* promoter and termed *cis* element IR-X. Schemes of these promoters are depicted in Fig. 2A. The modified and native promoters were fused to the Aspergillus niger *goxA* gene, which is an established reporter system in Trichoderma species (18–21), and subsequently transformed into T. reesei QM6aΔ*tmus53*Δ*pyr4* (22). Using the reestablishment of *pyr4* as a marker allowed the selection of uridine prototrophic strains. Correct genotypic modification, i.e., the insertion of one copy at the *pyr4* locus, was tested by PCR and Southern blot hybridization (see Fig. S1A and B in the supplemental material). To validate the experimental design and as a first approach, we investigated these strains by a carbon source replacement experiment.

Pc_WT, the strain expressing *goxA* under the native *cbh1* promoter, had basal GoxA activity on medium without any carbon source and on glycerol (Fig. 2B). While we observed CCR on d-glucose, we detected slight induction on a low concentration of d-xylose and strong induction on sophorose (Fig. 2B). The results of the GoxA assays reproduce already published quantitative PCR (qPCR) data on *cbh1* from similar carbon source replacement experiments (2). Together, these results confirmed the suitability of this reporter assay for our research objectives. The deletion of *cis* element X (strain Pc_ΔX) released the *cbh1* promoter slightly from CCR. With regard to inducing conditions, the deletion had no effect using d-xylose and led to a minor decrease of activity on sophorose (Fig. 2B). The *cis* element exchange in strain Pc_X-IR led to a general increase of *cbh1* promoter activity regardless of which condition (i.e., carbon source) was used (Fig. 2B). In testing the *xyn1* promoter, the native one in strain Px_WT was only induced on a low concentration of d-xylose (Fig. 2B). Induction on sophorose was not observed. Deletion of the *cis* element IR in strain Px_ΔIR led to a loss of inducibility on low concentrations of d-xylose (Fig. 2B). This loss could also be observed in strain Px_IR-X, bearing the motif exchange IR for X in the *xyn1* promoter. Neither was induction by sophorose observed in this strain (Fig. 2B).

As mentioned above, the strain Pc_WT behaved as expected on all carbon sources. The deletion of the *cis* element X only led to minor changes, i.e., decreased GoxA expression under inducing conditions and a slight release from CCR under repressing conditions. Otherwise, the *cis* element exchange X-IR led generally to higher promoter activity; for example, 3.3-times-higher GoxA activity under inducing conditions than with the native promoter. The regulation of the *xyn1* promoter is much tighter. Under several conditions, the GoxA activity was below quantification or detection limits (Fig. 2B). d-Xylose-mediated inducibility was lost when the *cis* element IR was absent, and the *cis* element X could not restore any inducibility. Therefore, we conclude that the *cis* element IR is necessary for inducibility of the *xyn1* promoter on d-xylose, while the *cis* element X does not mediate sophorose inducibility in either of the two promoters. Interestingly, the presence of *cis* element IR in the *cbh1* promoter led to a generally stronger promoter. These results raised the question of whether *cis* element modification allows the use of alternative carbon sources for the same or improved promoter activation as reached with known inducers.

### The *cis* element IR mediates strong inducibility of the *cbh1* promoter by wheat straw.

**FIG 3:**
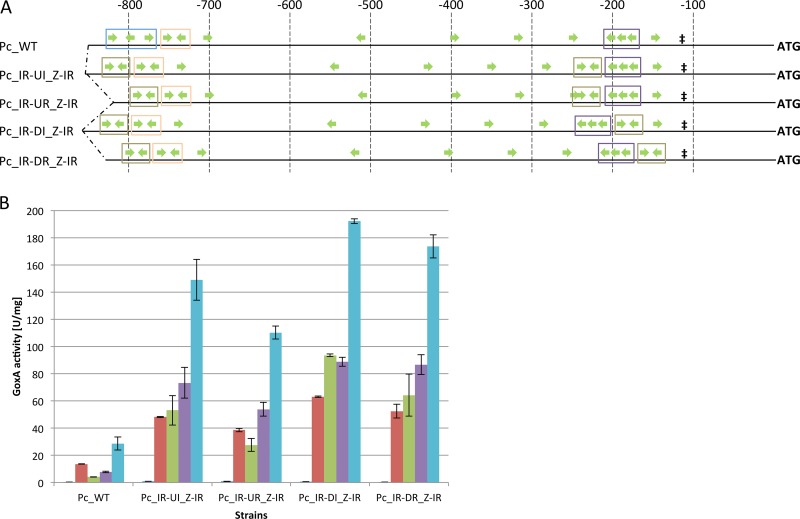
GoxA activities of strains bearing modified *cbh1* promoters after direct cultivation. (A) Green arrows indicate XBS and their orientations, ‡ indicates the TATA signal, and ATG represents the start codon of the reporter gene *goxA*. Blue boxes indicate *cis* element Z, orange boxes indicate *cis* element Y, purple boxes indicate *cis* element X, and brown boxes indicate *cis* element IR. Numbers on top indicate the distance from the ATG in base pairs. (B) Indicated strains were grown on MA medium with 1% glycerol (dark blue bars), lactose (red bars), xylan (green bars), carboxymethylcellulose (purple bars), or pretreated wheat straw (light blue bars). GoxA activity is given in relation to biomass (NaOH-soluble protein). The mean values shown are calculated from the results of biological duplicates and technical triplicates. Error bars indicate standard deviations.

The results showing that the *cbh1* promoter strength can be increased by the insertion of the *cis* element IR prompted us to study the impact of this *cis* element in detail. We investigated four different promoter versions, which are depicted in Fig. 3A. All four approaches have in common that the *cis* element Z was exchanged for the *cis* element IR (this exchange is termed Z-IR). Besides this, an additional *cis* element IR was inserted either downstream (termed IR-DI) or upstream (termed IR-UI) from the *cis* element X (maintaining the nucleotide distances between the *cis* elements X and IR and the surrounding elements) or the *cis* element IR was used to replace the sequences downstream (IR-DR) or upstream (IR-UR) from *cis* element X (Fig. 3A). Since CBHI is the T. reesei enzyme that is most relevant for industry-scale production, we chose to perform cultivations, which are more closely related to biotechnological approaches than carbon source replacement experiments. Consequently, we cultivated the recombinant strains directly on medium containing glycerol (considered a neutral carbon source), xylan (a complex substrate that preferentially induces xylanase expression), carboxymethylcellulose (CMC; pure, soluble cellulose that preferentially induces cellulase expression), lactose (induces cellulase expression), or pretreated wheat straw (a very complex, renewable so-called “biowaste”) as the carbon source.

GoxA activity was increased among all strains carrying a modified *cbh1* promoter on all carbon sources compared to the GoxA activity in the strain bearing the native promoter (Fig. 3B). Surprisingly, the highest levels were observed on pretreated wheat straw, followed by CMC, xylan, and lactose as substrates (Fig. 3B). In addition to the promoter strength, the inducibility, i.e., the GoxA activity obtained on a certain carbon source compared to the GoxA activity on glycerol, was affected. The levels of inducibility of the native *cbh1* promoter (strain Pc_WT) were 46.9 ± 1.8 (mean ± standard deviation), 14.1 ± 0.6, 27.1 ± 2.3, and 99 ± 16.8 with lactose, xylan, CMC, and pretreated wheat straw, respectively, as the substrate. The biggest impact on promoter inducibility could be observed in strain Pc_IR-DR_Z-IR, for which the respective values increased to 260 ± 71.8, 318.8 ± 112.8, 430 ± 116.6, and 861.4 ± 226.1.

Considering that the strong *cbh1* promoter has a notably higher number of XBS than the weaker *xyn1* promoter and that most of the strains investigated so far gained XBS by modifying the promoter, we decided to examine the question of whether the increase in the number of XBS alone causes the observed impacts on the inducibility and/or the strength of the *cbh1* promoter.

### Increasing distances of *cis* elements to TSS correlate positively with promoter inducibility.

**FIG 4:**
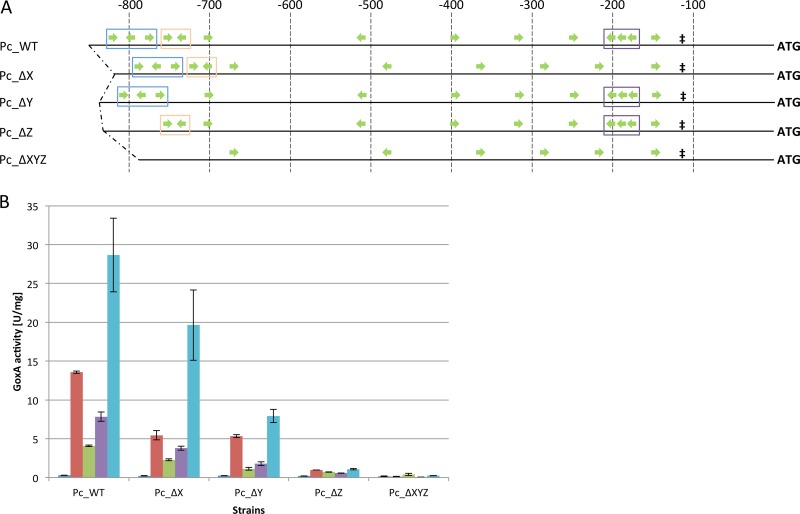
GoxA activities of strains bearing modified *cbh1* promoters after direct cultivation. (A) Green arrows indicate XBS and their orientations, ‡ indicates the TATA signal, and ATG represents the start codon of the reporter gene *goxA*. Blue boxes indicate *cis* element Z, orange boxes indicate *cis* element Y, purple boxes indicate *cis* element X, and brown boxes indicate *cis* element IR. Numbers on top indicate the distance from the ATG in base pairs. (B) Indicated strains were grown on MA medium with 1% glycerol (dark blue bars), lactose (red bars), xylan (green bars), carboxymethylcellulose (purple bars), or pretreated wheat straw (light blue bars). GoxA activity is given in relation to biomass (NaOH-soluble protein). The mean values shown are calculated from the results of biological duplicates and technical triplicates. Error bars indicate standard deviations.

In order to investigate the influence of the number of XBS on the *cbh1* promoter activity, we deleted the XBS from each *cis* element (termed ΔX, ΔY, and ΔZ, respectively) and all of them together (termed ΔXYZ). Figure 4A displays the modified promoters and provides the names of the corresponding strains that were investigated.

For all respective strains, the promoter activity decreased; however, it decreased to different extents depending on the particular *cis* element in which the XBS were deleted (Fig. 4B). What we observed was distinct losses of activity related to the lack of the native *cis* element in the following order: X, Y, Z (Fig. 4B). Notably, the pattern of induction (related to the carbon source used) remained the same. Finally, the strain that lacks the native *cis* element Z was only minimally inducible, and inducibility was completely lost in strain Pc_ΔXYZ, in which the XBS from all three *cis* elements are deleted (Fig. 4B). Obviously, the decrease in inducibility correlated directly with the distance from the *cis* element to the TSS of the reporter: the further upstream the *cis* element was located, the stronger was the decrease of promoter activity in the case of deletion of its XBS. Importantly, the strain lacking the native *cis* element Z had only a minimally higher GoxA activity than the strain lacking all three native elements, suggesting that just the number of XBS has a minor role compared to their position. Interestingly, the GoxA activity on the neutral carbon source glycerol is hardly affected by the deletion of XBS in any of these *cis* elements (Fig. 4B), which indicates a small impact of XBS in basal protein expression from this promoter. As we saw only a minor influence of the sheer number of XBS on promoter activity but a clear distance dependency, we decided to investigate whether the arrangement of *cis* elements can even overrule the number of XBS.

### The configuration of *cis* elements is predominant over the sheer number of XBS.

**FIG 5:**
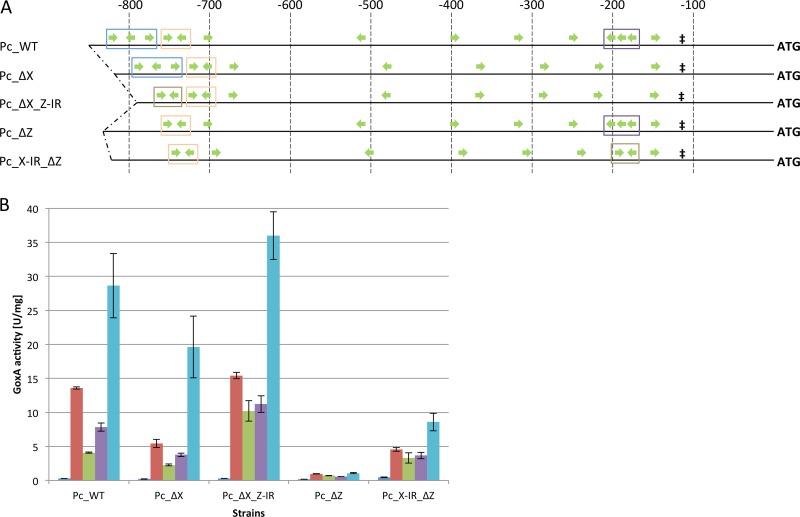
GoxA activities of strains bearing modified *cbh1* promoters after direct cultivation. (A) Green arrows indicate XBS and their orientations, ‡ indicates the TATA signal, and ATG represents the start codon of the reporter gene *goxA*. Blue boxes indicate *cis* element Z, orange boxes indicate *cis* element Y, purple boxes indicate *cis* element X, and brown boxes indicate *cis* element IR. Numbers on top indicate the distance from the ATG in base pairs. (B) Indicated strains were grown on MA medium with 1% glycerol (dark blue bars), lactose (red bars), xylan (green bars), carboxymethylcellulose (purple bars), or pretreated wheat straw (light blue bars). GoxA activity is given in relation to biomass (NaOH-soluble protein). The mean values shown are calculated from the results of biological duplicates and technical triplicates. Error bars indicate standard deviations.

Concerning the impact of *cis* elements on promoter activity, we aimed to identify the exact hierarchy of the following properties: number of XBS, arrangement of XBS, and distance of XBS from TSS. Therefore, in strain Pc_ΔX, *cis* element Z was exchanged for *cis* element IR (resulting in strain Pc_ΔX_Z-IR), and in strain Pc_ΔZ, *cis* element X was replaced with *cis* element IR (resulting in strain Pc_X-IR_ΔZ) (Fig. 5A).

Comparing the GoxA activities of Pc_ΔX and Pc_ΔX_Z-IR with that of the strain bearing the native promoter, we observed a similar induction pattern (Fig. 5B). However, strain Pc_ΔX_Z-IR, which bears four fewer XBS than the native *cbh1* promoter, had higher activity than strain Pc_WT. The highest increase in GoxA levels was observed on xylan (2.51-fold ± 0.37-fold higher than in Pc_WT). Otherwise, strain Pc_ΔX, which has one more XBS than Pc_ΔX_Z-IR, could not reach the levels of Pc_WT (Fig. 5B). A similar observation was made in comparing Pc_ΔZ and Pc_X-IR_ΔZ: higher GoxA expression was observed when *cis* element X was exchanged for IR, even though the XBS number was lower. However, Pc_X-IR_ΔZ also did not reach the GoxA levels of Pc_WT. Finally, the best performing strain was Pc_ΔX_Z-IR, which has a rather low number of XBS. Interestingly, these XBS are located quite distant from the TSS. The promoter of strain Pc_X-IR_ΔZ, which bears the same number of XBS and the same *cis* elements but with the IR located close to the TSS, was much weaker. Besides this, GoxA expression on glycerol was again only slightly influenced by the promoter modifications (Fig. 5B).

These results suggest that the three factors arrangement of *cis* element, distance of *cis* element to TSS, and number of XBS clearly have a descending order in their influence on promoter inducibility. Finally, we aimed to get a more complete picture of the *cbh1* promoter variants by combining all available promoter fragments (i.e., modifications in the 5′ region and the 3′ region).

### The results for all possible *cis* element variations endorse the previous findings.

**FIG 6:**
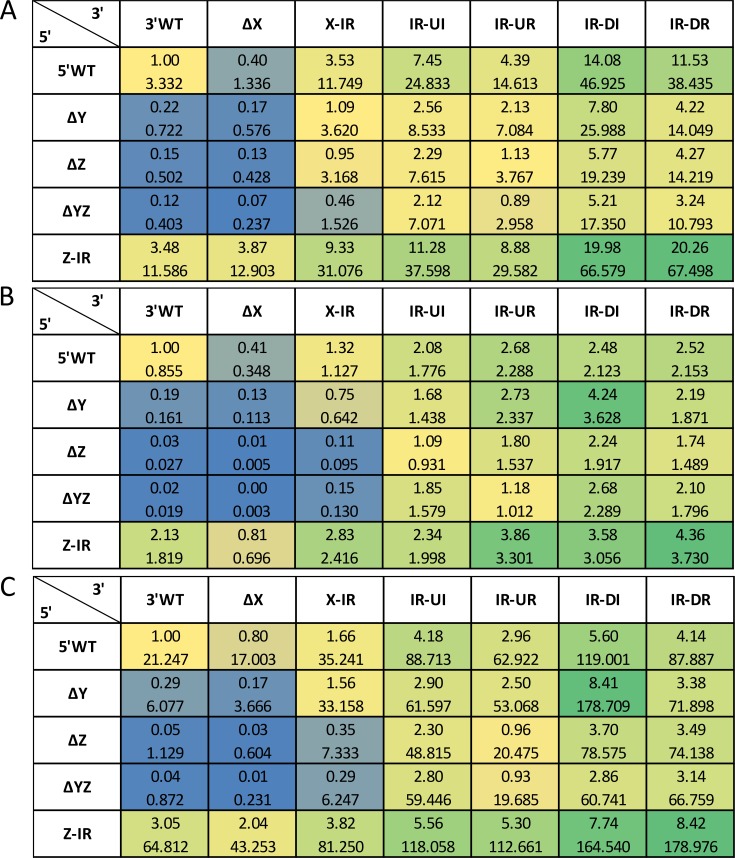
GoxA activities of strains bearing combinations of modified *cbh1* promoter fragments after direct cultivation. Thirty-five recombinant strains bearing different combinations of 3′ and 5′ fragments of the *cbh1* promoter (compare Table 3) were grown on MA medium with 1% xylan (A), lactose (42 h cultivation time) (B), or pretreated wheat straw (C). The top value in each table cell gives the GoxA activity relative to that in strain Pc_WT. The bottom value in each table cell gives the absolute GoxA activity in relation to biomass, i.e., U/mg NaOH-soluble protein (A, C) or U/mg dry weight (B). Color key: the GoxA activity values of Pc_WT are highlighted in yellow; blue indicates lower and green indicates higher GoxA activities. The mean values shown are calculated from the results of technical triplicates. Error bars indicate standard deviations.

To obtain a full picture of the *cis* element influence on the *cbh1* promoter, we investigated *cis* element variations within the *cbh1* promoter by combining all available 5′ and 3′ fragments (compare Table 2 below and Fig. 6). The respective recombinant strains were grown on xylan, lactose, and pretreated wheat straw.

On xylan, the GoxA expression of the majority of strains carrying a *cbh1* promoter containing an additional *cis* element IR in the vicinity of *cis* element X (IR-UI, IR-UR, IR-DI, and IR-DR) was induced (Fig. 6A). In strains in which the *cis* element Z is exchanged for *cis* element IR (Z-IR), the induction was even stronger. The deletion of XBS in the *cis* elements led to a distance-dependent decrease in promoter activity (ΔX, ΔY, ΔZ, ΔYZ, and ΔXYZ). For the combinations of XBS deletions and the addition of *cis* element IR, we found a clear potential of the latter to compensate for the loss of XBS (Fig. 6A).

Similar observations were made on lactose (Fig. 6B). The addition of *cis* element IR and/or *cis* element exchanges increased the strength of the promoter, and the deletion of XBS from *cis* elements decreased the strength of the promoter in a manner dependent on the distance from the TSS. Overall, the effects were a bit less pronounced than on xylan.

Finally, strains were cultured on pretreated wheat straw and analyzed for *cbh1* promoter strength (Fig. 6C). The addition of *cis* element IR and/or *cis* element exchanges increased the strength of the respective *cbh1* promoter and led to very high levels of GoxA activity. Deletions of XBS from *cis* elements led to decreases in promoter activity. Again, the combinations of XBS deletions and the addition of *cis* element IR had a tendency to compensate for the loss of the native *cis* elements.

Summarizing the results from the latter detailed approach, which allowed us to investigate a greater number of variations of *cis* elements in the *cbh1* promoter, this demonstrated the strong influence of the *cis* element IR on promoter strength. The comparison of the variations supported the finding that the number of XBS has only a secondary effect on promoter strength. All combinations of *cis* element deletions pointed to decreased promoter strength with increased distance from the TSS. Notably, the GoxA activity patterns of the strains carrying modified *cbh1* promoter constructs are very constant when comparing different substrates.

## DISCUSSION

In the past, relevant regions and motifs for the regulation of the *xyn1* gene were identified (5, 6). In this study, we present results demonstrating that the previously identified *cis* element IR, an inverted repeat of XBS, conveys transcriptional induction on xylan and wheat straw. We studied the role of this *cis* element by deleting only this specific motif and not by classic deletion analysis of the promoter. Phenotypic analysis was initially performed by carbon source replacement experiments because growth effects can be excluded and this allowed us to focus on the impact of a single carbon source on the promoter activity.

As Xyr1 is the essential transactivator not only of the *xyn1* gene but also of the industrially more relevant *cbh1* gene (4), we extended our study to investigate the *cis* element IR in both promoters. In the case of the *cbh1* promoter, Ilmén and coworkers performed a first deletion mapping (23) and found that CCR was abolished when a sequence upstream from −500 bp was removed and that the modification of a DNA motif at −720 bp was enough to trigger derepression. Furthermore, they found that sophorose induction still worked when the promoter sequence upstream from position −161 bp was removed. Years later, two reports on the *cbh1* promoter were published by Liu et al. (15) and Zou et al. (16): the first study inserted the sequence stretch from −620 to −820 bp of the *cbh1* promoter multiple times in the 5′ end of this promoter, leading to increased reporter activity. In the other study, Cre1-binding sites were exchanged with binding sites for Ace2 or the Hap2/3/5 complex, which also resulted in increased reporter gene expression. In both studies, the promoter-reporter constructs were randomly integrated into the genomes of Rut-C30 descendant strains. In the present study, we used targeted integration at the *pyr4* locus of the wild-type T. reesei strain QM6a (22) in order to ensure genotypic comparability of all recombinant strains. Besides this, we specifically modified the *cis* elements instead of deleting or inserting longer sequence stretches. This strategy has the advantage that the core promoter does not become shorter and the effects of unknown regulatory sequences are not observed unintentionally.

In Saccharomyces cerevisiae, Gal4 binds inverted repeats of the sequence 5′-CGG(N_11_)CCG-3′ as a dimer in the upstream activating sequence (UAS) of the *gal1* gene (24). The Gal4 DNA-binding consensus is very conservative concerning the spacer between the inverted repeats. In the present study, we investigated binding sites that are contacted by Xyr1, a Gal4-like transcription factor. Xyr1 is also supposed to bind DNA as a dimer; however, the spacer between the target sequences does not seem to be restricted (25). As Ace1 contacts DNA sequences similar to those contacted by Xyr1, there could also be heterodimerization involved in the transcriptional regulation. Strikingly, in the present study, the exchange of a direct for an inverted repeat of XBS caused different substances to become strong inducers of gene expression (e.g., wheat straw in the case of the *cbh1* gene). From a scientific point of view, it is an interesting consideration that genes whose expression is triggered by different inducing substances, although activated by the same transactivator, respond to another inducer as a result of another arrangement of the binding sites of the transactivator. And from an application-oriented point of view, this finding could support the reduction of operating costs by allowing cheaper, more easily available or more sustainable inducer substances to be used.

Murphy and coworkers investigated the impact of the position of *cis* elements by modifying the *gal1* promoter in S. cerevisiae (26). They inserted one to three *tet*O_2_ operators between the TATA signal and the TSS and analyzed the influence of the different promoters on yeast eGFP expression. This approach involved the addition of different concentrations of an inhibitor of the TetR repressor protein. When the *tet*O_2_ operator was inserted near the TSS, the promoter was active even at low concentrations of the TetR inhibitor. Otherwise, when the *tet*O_2_ operator was inserted near the TATA signal, the promoter became active only at higher concentrations of the inhibitor. Even if the strength of the promoter did not change significantly, the inducibility of the systems concerning dependence on dosage was modified. In our study, modifying the positions of relevant *cis* elements enhanced the general strength and the inducibility of the promoter. This might be due to the fact that we changed the positions of binding sites for a transactivator, while in S. cerevisiae, a repressor/inhibitor system was investigated. Blazeck and coworkers investigated hybrid promoters in S. cerevisiae (27). They used synthetic promoters composed of five different core promoters (of the *cyc*, *gal1*, *gpd*, *leu3*, and *tef* genes) and fused the UASs of the *cit1*, *clb2*, *gal1*, and *tef* genes to their 5′ ends. The hybrid promoter combining the *leu3* core promoter and three *cbl2* UASs was 10 times stronger than the native *leu3* promoter. They also complemented the *leu3* promoter with inhibitory regions or Gal4p-binding sites. Altogether, they observed only active induction in the case of promoter-reporter strains containing the Gal4p-binding sites. From these and our findings, it can be assumed that for the aim of increasing promoter strength, the targeting of binding sites for the respective transactivator is the most straightforward strategy.

It should be noted that the reporter gene *goxA*, which was used in this study, originates from another filamentous fungus (namely, A. niger). Even if we cannot safely assume that any other heterologous target protein can be produced with the same efficiency as GoxA, the strategy of using the *cbh1* promoter and T. reesei as the host organism suggests itself as a promising one. In particular, the option to use wheat straw as a cheap and sustainable substrate to provide high yields of a target protein adds attractiveness to this approach.

## MATERIALS AND METHODS

### Strains and cultivation conditions.

T. reesei strains QM6aΔ*tmus53*Δ*pyr4* (28), QM6a Pc_WT (33) (termed QM6aΔ*tmus53*_pc1g therein), QM6a Pc_X-IR (33) (termed QM6aΔ*tmus53*_pcxg therein), and all other strains used in this study were maintained on malt extract agar at 30°C. Mandels-Andreotti (MA) medium (29) without peptone was used as a minimal medium. All strains were derived from QM6aΔ*tmus53*Δ*pyr4* (28). An overview is provided in Table S1 in the supplemental material.

For carbon source replacement experiments, mycelia were precultured in 200 ml MA medium supplemented with 0.1% peptone and 1% (wt/vol) glycerol as the sole carbon source on a rotary shaker at 180 rpm at 30°C for 24 h. A total of 10^9^ conidia per liter (final concentration) was used as the inoculum. Pregrown mycelia were washed, and equal amounts were resuspended in 20 ml MA medium containing either no carbon source or 1% (wt/vol) glycerol, 1% (wt/vol) d-glucose, 0.5 mM d-xylose, 1% (wt/vol) d-xylose, or 1.5 mM sophorose as the sole carbon source and were incubated for 8 h. Culture supernatants were derived from biological duplicates.

For direct cultivation experiments, mycelia were cultured in 20 ml MA medium supplemented with 0.1% peptone and either 1% (wt/vol) glycerol, 1% (wt/vol) lactose, 1% xylan (wt/vol) (Lenzing AG, Lenzing, Austria), 1% (wt/vol) carboxymethylcellulose, or 1% (wt/vol) pretreated wheat straw as the sole carbon source on a rotary shaker at 180 rpm at 30°C for 48 h if not indicated otherwise in the figure legends. The mycelia growing on pretreated wheat straw were incubated for 72 h. Samples were derived from biological duplicates.

Escherichia coli strain Top10 (Invitrogen, Life Technologies, Paisley, United Kingdom) was used for all cloning purposes throughout this study and maintained on LB at 37°C. If applicable, ampicillin or spectinomycin was added to a final concentration of 100 mg/ml or 100 mg/ml, respectively.

### Plasmid construction.

PCRs for cloning purposes were performed with Phusion high-fidelity DNA polymerase (Thermo Fisher Scientific, Waltham, MA, USA) according to the manufacturer's instructions. All primers used are listed in Table 1.

**TABLE 1:** List of primers used

Name	Sequence (5′ → 3′)
5pyr4_fwd3	CCAGACGGTGATTCACATATACG
goxa_fw_Bam	GGATCCATGCAGACTCTCCTTGTGAGCTCG
goxa_rv_Bcu-Nhe	GCTAGCACTAGTTCACTGCATGGAAGCATAATCTTCC
pcbh1_fw_Cfr	CCCGGGACTGGAAAATACAAACCAATGGCTAAAAG
pcbh1_rv_Bam_Nhe	GCTAGCATTAGGATCCGATGCGCAGTCCGCGG
pcbh1_delta_x_rv_(soe)	CGTTTAATGAGCTTCTCTTTCTATTCGAAACC
pcbh1_delta_x_rv_(soe)	AAAGAGAAGCTCATTAAACGGAATGAGCTAGTAGG
pxyn1_fw_Cfr	CCCGGGCTGCAGCAAATGGCCTCAAGC
pxyn1_rv_Bam-Nhe	GCTAGCATGCGGATCCGATGATTATTTGTGCGTGTTTTCCTTG
pxyn1_delta_ir_rv_(soe)	TCTGGGGTGCATCCTGCCAATCAAGTCAAGGGGC
pxyn1_delta_ir_fw_(soe)	TTGATTGGCAGGATGCACCCCAGATCTG
pxyn1_ir-x_fw_(soe)	TTAGCCAAGAACAATAGCCGATAAAGATAGCCGGATGCACCCCAGATCTG
pxyn1_ir-x_rv_(soe)	GCTATCTTTATCGGCTATTGTTCTTGGCTAATGCCAATCAAGTCAAGGGG
pyr4_3fwd	AGACGAGGACCAGCAGACC
Tpyr4_rev2	CAGGAAGCTCAGCGTCGAG

For the construction of pDK/P*xyn1* (P*xyn1*::*goxA*; GenBank accession number KY780443), a PCR product was created with primers pxyn1_fw_Cfr and pxyn1_rv_Bam_Nhe using chromosomal DNA from T. reesei QM6a as the template. The fragment was inserted into the EcoRV-digested vector pJET1.2 (Thermo Fisher Scientific), yielding pDK/P*xyn1*. pDK/GoxA was created analogously with primers goxa_fwd-Bam and goxa_rv_Bcu-Nhe, using pLW-WT (30) as the template in the PCR. The *goxA* structural gene was inserted into pDK/P*xyn1* using BamHI and NheI digestion of pDK/GoxA, resulting in pDK/P*xyn1*::*goxA*. The promoter-reporter construct was excised using BcuI and Cfr9I and ligated into pCD-RPyr4T (22) (GenBank accession number KT176093), which was digested beforehand using BcuI and Kpn2I, yielding pRPyr/P*xyn1*::*goxA*.

For the construction of pDK/P*xyn1*_ΔIR (P*xyn1*_ΔIR::*goxA*; GenBank accession number KY780444), two PCR products were created with primers pxyn1_fw_Cfr and pxyn1_delta_ir_rv_(soe), as well as pxyn1_delta_ir_fw_(soe) and pxyn1_rv_Bam-Nhe, using chromosomal DNA from T. reesei QM6a as the template. Both fragments were purified using an agarose gel extraction kit (Thermo Fisher Scientific). Thereafter, a splicing by overlap extension (SOE) PCR was performed with primers pxyn1_fw_Cfr and pxyn1_rv_Bam-Nhe, using the two fragments as the template. The resulting fragment was inserted into the EcoRV-digested vector pJET1.2 (Thermo Fisher Scientific), yielding pDK/P*xyn1*_ΔIR. Plasmids pDK/P*xyn1*_ΔIR::*goxA* and pRPyr/P*xyn1*_ΔIR::*goxA* were created analogously to pDK/P*xyn1*::*goxA* and pRPyr/P*xyn1*::*goxA*.

For the construction of pDK/P*xyn1*_IR-X (P*xyn1*_IR-X::*goxA*; GenBank accession number KY780445), two PCR products were created with primers pxyn1_fw_Cfr and pxyn1_ir-xr_rv_(soe), as well as pxyn1_ir-x_fw_(soe) and pxyn1_rv_Bam-Nhe, using chromosomal DNA from T. reesei QM6a as the template. Both fragments were purified using an agarose gel extraction kit (Thermo Fisher Scientific). Thereafter, an SOE PCR was performed with primers pxyn1_fw_Cfr and pxyn1_rv_Bam-Nhe using the two fragments as the template. The resulting fragment was inserted into the EcoRV-digested vector pJET1.2 (Thermo Fisher Scientific), yielding pDK/P*xyn1*_IR-X. Plasmids pDK/P*xyn1*_IR-X::*goxA* and pRPyr/P*xyn1*_IR-X::*goxA* were created analogously to pDK/P*xyn1*::*goxA* and pRPyr/P*xyn1*::*goxA*.

For the construction of pDK/P*cbh1*_ΔX, two PCR products were created with primers pcbh1_fw_Cfr and pcbh1_delta_x_rv_(soe), as well as pcbh1_delta_x_fw_(soe) and pcbh1_rv_Bam-Nhe, using chromosomal DNA from T. reesei QM6a as the template. Both fragments were purified using an agarose gel extraction kit (Thermo Fisher Scientific). Thereafter, an SOE PCR was performed with primers pcbh1_fw_Cfr and pcbh1_rv_Bam-Nhe using the two fragments as the template. The resulting fragment was inserted into the EcoRV-digested vector pJET1.2 (Thermo Fisher Scientific), yielding pDK/P*cbh1*_ΔX. Plasmids pDK/P*cbh1*_ΔX::*goxA* and pRPyr/P*cbh1*_ΔX::*goxA* were created analogously to pDK/P*cbh1*_ΔX::*goxA* and pRPyr/P*xyn1*::*goxA*.

The *cbh1* promoter can be cut with Bsp120I, yielding a 5′ and a 3′ fragment; in the case of fragments P*cbh1*_WT, P*cbh1*_X-IR (33), and P*cbh1*_ΔX, this digestion yields the 5′ fragment 5′WT, as well as 3′ fragments 3′WT, 3′X-IR, and 3′ΔX, respectively. Additional promoter fragments were obtained by custom order from GeneArt (Thermo Fisher Scientific). Table 2 provides an overview of the promoter fragments used and the plasmids carrying them.

**TABLE 2:** Overview of promoter fragments used

Promoter fragment	Plasmid (reference)	Remarks
ΔY	pMA-T/P*cbh1*_ΔY^*a*^	p*cbh1* 5′ fragments resulting from Cfr9I/Bsp120I double digest of the respective plasmid
ΔZ	pMA-T/P*cbh1*_ΔZ^*a*^	
ΔYZ	pMA-T/P*cbh1*_ΔYZ^*a*^	
Z-IR	pMA-T/P*cbh1*_Z-IR^*a*^	
5′WT	pDK/P*cbh1*_WT (33)	
IR-UI	pMA-T/P*cbh1*_IR-UI^*a*^	p*cbh1* 3′ fragments resulting from Bsp120I/BamHI double digest of the respective plasmid
IR-UR	pMA-T/P*cbh1*_IR-UR^*a*^	
IR-DI	pMA-T/P*cbh1*_IR-DI^*a*^	
IR-DR	pMA-T/P*cbh1*_IR-DR^*a*^	
3′WT	pDK/P*cbh1*_WT (33)	
X-IR	pDK/P*cbh1*_X-IR (33)	
ΔX	pDK/P*cbh1*_ΔX	

a Plasmid was ordered from GeneArt (Thermo Fisher Scientific).

Table 3 provides an overview of the different combinations of native and modified 5′ and 3′ fragments of the *cbh1* promoter. In the case of ΔX, the whole *cis* element was deleted from the *cbh1* promoter, and in the case of ΔY and ΔZ, just the XBS were destroyed.

**TABLE 3:** Overview of the *cbh1* promoter-reporter constructs and corresponding recombinant T. reesei strains

5′ fragment used	3′ fragment used^*a*^						
	WT	ΔX	X-IR	IR-UI	IR-UR	IR-DI	IR-DR
WT	P*cbh1*::*goxA*	P*cbh1*_ΔX::*goxA*	P*cbh1*_X-IR::*goxA*	P*cbh1*_IR-UI::*goxA*	P*cbh1*_IR-UR::*goxA*	P*cbh1*_IR-DI::*goxA*	P*cbh1*_IR-DR::*goxA*
	Pc_WT^*b*^	Pc_ΔX^*b*^	Pc_X-IR^*b*^	Pc_IR-UI	Pc_IR-UR	Pc_IR-DI	Pc_IR-DR
	KY780446	KY780447	KY780448	KY780449	KY780450	KY780451	KY780452
ΔY	P*cbh1*_ΔY::*goxA*	P*cbh1*_ΔXY::*goxA*	P*cbh1*_X-IR,ΔY::*goxA*	P*cbh1*_IR-UI,ΔY::*goxA*	P*cbh1*_IR-UR,ΔY::*goxA*	P*cbh1*_IR-DI,ΔY::*goxA*	P*cbh1*_IR-DR,ΔY::*goxA*
	Pc_ΔY^*c*^	Pc_ΔXY	Pc_X-IR_ΔY	Pc_IR-UI_ΔY	Pc_IR-UR_ΔY	Pc_IR-DI_ΔY	Pc_IR-DR_ΔY
	KY780453	KY780454	KY780455	KY780456	KY780457	KY780458	KY780459
ΔZ	P*cbh1*_ΔZ::*goxA*	P*cbh1*_ΔXZ::*goxA*	P*cbh1*_X-IR,ΔZ::*goxA*	P*cbh1*_IR-UI,ΔZ::*goxA*	P*cbh1*_IR-UR,ΔZ::*goxA*	P*cbh1*_IR-DI,ΔZ::*goxA*	P*cbh1*_IR-DR,ΔZ::*goxA*
	Pc_ΔZ^*c*^	Pc_ΔXZ	Pc_X-IR_ΔZ^*d*^	Pc_IR-UI_ΔZ	Pc_IR-UR_ΔZ	Pc_IR-DI_ΔZ	Pc_IR-DR_ΔZ
	KY780460	KY780461	KY780462	KY780463	KY780464	KY780465	KY780466
ΔYZ	P*cbh1*_ΔYZ::*goxA*	P*cbh1*_ΔXYZ::*goxA*	P*cbh1*_X-IR,ΔYZ::*goxA*	P*cbh1*_IR-UI,ΔYZ::*goxA*	P*cbh1*_IR-UR,ΔYZ::*goxA*	P*cbh1*_IR-DI, ΔYZ::*goxA*	P*cbh1*_IR-DR, ΔYZ::*goxA*
	Pc_ΔYZ	Pc_ΔXYZ^*c*^	Pc_X-IR_ΔYZ	Pc_IR-UI_ΔYZ	Pc_IR-UR_ΔYZ	Pc_IR-DI_ΔYZ	Pc_IR-DR_ΔYZ
	KY780467	KY780468	KY780469	KY780470	KY780471	KY780472	KY780473
Z-IR	P*cbh1*_Z-IR::*goxA*	P*cbh1*_ΔX,Z-IR::*goxA*	P*cbh1*_X-IR,Z-IR::*goxA*	P*cbh1*_IR-UI,Z-IR::*goxA*	P*cbh1*_IR-UR,Z-IR::*goxA*	P*cbh1*_IR-DI,Z-IR::*goxA*	P*cbh1*_IR-DR, Z-IR::*goxA*
	Pc_Z-IR	Pc_ΔX_Z-IR^*d*^	Pc_X-IR_Z-IR	Pc_IR-UI_Z-IR^*e*^	Pc_IR-UR_Z-IR^*e*^	Pc_IR-DI_Z-IR^*e*^	Pc_IR-DR_Z-IR^*e*^
	KY780474	KY780475	KY780476	KY780477	KY780478	KY780479	KY780480

a Promoter-reporter constructs were obtained by combining the 3′ fragments and 5′ fragments. Table provides the name of the promoter-reporter construct (upper term) and the GenBank accession number of the respective construct (lower term) that was transformed into the fungal genome, yielding the respective recombinant strain (middle term).

b A promoter scheme is provided in Fig. 2.

c A promoter scheme is provided in Fig. 4.

d A promoter scheme is provided in Fig. 5.

e A promoter scheme is provided in Fig. 3.

In a first step, plasmids pDK/P*cbh1*_IR-UI::*goxA*, pDK/P*cbh1*_IR-UR::*goxA*, pDK/P*cbh1*_IR-DI::*goxA*, and pDK/P*cbh1*_IR-DR::*goxA* were created. The pMA-T plasmids carrying the corresponding 3′ fragments and pDK/P*cbh1*_WT::*goxA* (33) were cut using Bsp120I and BamHI. The 3′ fragments and the target vector were gel eluted and ligated, yielding the respective pDK/P*cbh1*_*::*goxA* constructs (where * is a “wild card”). In order to create all other constructs, pMA-T/P*cbh1*_ΔY, pMA-T/P*cbh1*_ΔZ, pMA-T/P*cbh1*_ΔYZ, and pMA-T/P*cbh1*_Z-IR and pDK/P*cbh1*_WT::*goxA*, pDK/P*cbh1*_ΔX::*goxA*, pDK/P*cbh1*_IR-UI::*goxA*, pDK/P*cbh1*_IR-UR::*goxA*, pDK/P*cbh1*_IR-DI::*goxA*, and pDK/P*cbh1*_IR-DR::*goxA* were digested using Cfr9I and Bsp120I. The fragments (originating from pMA-T plasmids) and the target vectors (digested pDK/P*cbh1*_*::*goxA* plasmids) were gel eluted. Each fragment was inserted into each vector to create the remaining pDK/P*cbh1*_*::*goxA* plasmids. Afterwards, each promoter-reporter construct was inserted into pCD/RPyr4T analogously to pRPyr/P*xyn1*::*goxA*, yielding a series of pRPyr/P*cbh1*_*::*goxA* plasmids.

All intermediate and final plasmids were sequenced by Microsynth (Balgach, Switzerland).

### Fungal transformation.

The protoplast transformation of T. reesei was performed as described by Gruber et al. (31). Typically, 50 μg of linearized plasmid DNA was used for transformation of 10^7^ protoplasts (in 200 μl). For selection of prototrophy, 500 μl of the transformation reaction mixture was added to 20 ml melted, warm (50°C) minimal medium agar containing 1.2 M sorbitol and 1% (wt/vol) d-glucose. This mixture was poured into sterile petri dishes, and after solidification, was incubated at 30°C for 3 to 7 days until colonies were visible. Table 3 provides an overview of the constructs that were transformed into the fungal genome and the resulting recombinant strains.

### Isolation of chromosomal DNA and PCR screening.

Chromosomal DNA was isolated from fungal mycelium by grinding in liquid nitrogen followed by a phenol-chloroform extraction (31). RNA was degraded using RNase A (Thermo Fisher Scientific). DNA was precipitated with isopropanol, washed with 70% (vol/vol) ethanol, and dissolved in 100 μl sterile bidistilled H_2_O. For testing the genotype, 10 ng of chromosomal DNA was used as the template in a 25-μl PCR mixture using GoTaq G2 polymerase (Promega, Madison, WI, USA) according to the manufacturer's instructions. All primers used are listed in Table 1. For subsequent agarose gel electrophoresis of DNA fragments, a GeneRuler 1-kb DNA ladder (Thermo Fisher Scientific) was applied to estimate fragment size.

### Southern blot analysis.

Fifteen micrograms of chromosomal DNA was digested with 30 U of SacII (New England BioLabs, Ipswich, MA, USA). The resulting DNA fragments were separated by electrophoresis on an 0.8% (wt/vol) agarose gel, denatured in 0.4 M NaOH, and transferred by capillary force onto a Biodyne B 0.45-μm nylon membrane (Pall Corporation, Port Washington, NY, USA) using 10× SSC (1× SSC is 0.15 M NaCl plus 0.015 M sodium citrate). A 1.5-μg quantity of biotinylated DNA probe was used for hybridization at 65°C overnight. Labeling of the probe was performed by using the Klenow fragment (exo^−^) (Thermo Fisher Scientific), random hexamer primers, and biotin-11-dUTP (Jena Bioscience, Jena, Germany). Signals were visualized by using poly-horseradish peroxidase (poly-HRP; Pierce) conjugated to streptavidin and ECL plus Western blotting substrate (Life Technologies) (both from Thermo Fisher Scientific) on a ChemiDoc MP (Bio-Rad Laboratories, Hercules, CA, USA).

### Glucose oxidase assay.

Glucose oxidase activity was assayed as described previously (18) using ABTS [2,2′-azino-di-(3ethyl-benzthiazoline sulfonate)] (Molekula Ltd., Gillingham, United Kingdom) and HRP (Sigma-Aldrich, St. Louis, MO, USA). One unit of activity is defined as the amount of enzyme that oxidizes 1 μmol of d-glucose per min at pH 5.8 and 25°C. Measurements were carried out in technical triplicates.

For determination of the biomass, the mycelium was separated from the culture supernatant by filtration using a predried piece of Miracloth and subsequently dried at 120°C for 24 h. Table S4 provides the biomass of strains grown on lactose. In the case of water-insoluble substrates, NaOH-soluble protein was determined and was used as an alternative reference to biomass.

### NaOH-soluble protein extraction.

Mycelia were separated from the culture supernatant by filtration (supernatant was used for glucose oxidase assays). Afterwards, each mycelium sample was washed using 30 ml deionized H_2_O and filtered again. The extraction of NaOH-soluble protein from the remaining biomass was performed as previously described (32). Twenty milliliters of 1 M NaOH was added to each mycelium sample and then fixed in a rotary shaker for 3 h. Samples were transferred into 1.5-ml tubes and centrifuged for 10 min at 20,000 × *g* to separate the extracted protein from the remaining solids. The protein concentration of the extract was determined using the Bio-Rad protein assay (Bio-Rad) against a bovine serum albumin (BSA) standard. Tables S2, S3, and S5 provide the concentrations of NaOH-soluble protein for T. reesei QM6a and recombinant strains cultivated on water-insoluble substrates.

### Accession number(s).

The data sets generated and analyzed during the current study are available in the GenBank repository (https://www.ncbi.nlm.nih.gov/GenBank/) under accession numbers KY780443 through KY780480; further information can be found in Table 3.

## Supplementary Material

Supplemental material
