# Supplementary material for: Influence of cis Element Arrangement on Promoter Strength in Trichoderma reesei
Source: Appl Environ Microbiol. 2017 Dec 15;84(1):e01742-17. doi: 10.1128/AEM.01742-17 (PMC5734013; doi:10.1128/AEM.01742-17)
Supplement: Supplemental material [file supp_84_1_e01742-17__index.html]

Influence of cis Element Arrangement on Promoter Strength in Trichoderma reesei — Supplemental material 

# Influence of *cis* Element Arrangement on Promoter Strength in Trichoderma reesei

## Supplemental material

- Supplemental file 1 -

  Strains and genotypic characterizations (Table S1); NaOH soluble protein details (Table S2); NaOH soluble protein of recombinant strains grown on xylan (Table S3); dry biomass of recombinant strains grown on lactose (Table S4); NaOH soluble protein of recombinant strains grown on pretreated wheat straw (Table S5); genotypic characterization of recombinant strains (Fig. S1).

  PDF, 2.0M
