# Supplementary material for: Influence of cis Element Arrangement on Promoter Strength in Trichoderma reesei
Source: Appl Environ Microbiol. 2017 Dec 15;84(1):e01742-17. doi: 10.1128/AEM.01742-17 (PMC5734013; doi:10.1128/AEM.01742-17)
Supplement: Supplemental material [file AEM.01742-17_zam001188235s1.pdf]

Table S1 Overview on used strains and their genotypic characterization

No.[1]	Strain name	Expected fragment sizes obtained by 5' PCR [bp][2]	Expected fragment sizes obtained by Southern blot analysis [bp][3]
1	Px_WT	2141	3039, 1496
2	Px_ΔIR	2119	3017, 1496
3	Px_IR-X	2151	3048, 1496
4	Pc_ΔX	2413	
5	Pc_ΔYZ	2415	
6	Pc_ΔX_Z-IR	2384	
7	Pc_X-IR_ΔY	2423	
8	Pc_IR-UI_ΔYZ	2450	
9	Pc_X-IR_ΔYZ	2405	
10	Pc_IR-UI	2480	
11	Pc_IR-UR	2445	
12	Pc_IR-DI	2484	
13	Pc_IR-DR	2455	
14	Pc_ΔY	2433	
15	Pc_ΔZ	2427	1496, 950
16	Pc_Z-IR	2416	
17	Pc_ΔXY	2401	
18	Pc_ΔXZ	2395	
19	Pc_ΔXYZ	2383	
20	Pc_X-IR_ΔZ	2417	
21	Pc_X-IR_Z-IR	2406	
22	Pc_IR-UI_ΔY	2468	
23	Pc_IR-UI_ΔZ	2462	
24	Pc_IR-UI_Z-IR	2451	
25	Pc_IR-UR_ΔY	2433	
26	Pc_IR-UR_ΔZ	2427	

27	Pc_IR-UR_ΔYZ	2415
28	Pc_IR-UR_Z-IR	2416
29	Pc_IR-DI_ΔY	2472
30	Pc_IR-DI_ΔZ	2466
31	Pc_IR-DI_ΔYZ	2454
32	Pc_IR-DI_Z-IR	2455
33	Pc_IR-DR_ΔY	2443
34	Pc_IR-DR_ΔZ	2437
35	Pc_IR-DR_ΔYZ	2425
36	Pc_IR-DR_Z-IR	2426

[1] The strain number is also used for the labelling of lanes for the genotypic characterization (PCR and Southern blot analysis) depicted in figure S1.

[2] The 5' PCR fragment sizes result from primers 5pyr_fwd3 and pcbh1_rv_bam-nhe for *cbh1* promoter-reporter constructs, and from primer 5pyr4_fwd3 and pxyn1_rv_bam-nhe for *xyn1* promoter-reporter constructs.

[3] The expected fragment sizes obtained after *Sac*II digest of chromosomal DNA by Southern blot hybridization using a labelled *goxA*-probe are given.

Table S2 Overview on NaOH soluble protein

Substrate / Strain	1% Glycerol		1% Lactose		1% Xylan		1% CMC		1% pretreated wheat straw	
**Pc_WT**	275 [1]	9 [2]	97	7	241	17	66	0	346	33
**Pc_ΔX**	299	1	118	6.	257	8	77	3	437	37
**Pc_ΔY**	294	5	8	6	220	12	63	2	465	25
**Pc_ΔZ**	284	5	118	10	242	4	70	4	407	38
**Pc_ΔXYZ**	274	10	123	16	218	8	64	4	368	6
**Pc_ΔX_Z-IR**	261	7	127	4	212	8	62	4	403	6
**Pc_X-IR_ΔZ**	277	3	106	3	223	14	64	2	377	28
**Pc_IR-UI_Z-IR**	259	4	100	1	204	3	63	0	387	17
**Pc_IR-UR_Z-IR**	275	4	101	2	214	10	65	4	350	6
**Pc_IR-DI_Z-IR**	259	3	102	2	199	1	59	0	345	19
**Pc_IR-DR_Z-IR**	263	2	111	12	199	7	59	1	253	21
**QM6a**	302	1	120	6	224	2	54	1	322	14

[1] The value on the left lane of each substrate gives the mean from biological duplicates in μg/ml.

[2] The value on the right lane of each substrate gives the standard deviation.

Table S3 Overview on NaOH soluble protein of recombinant strains[1] grown on xylan

	3' WT	ΔX	X-IR	IR-UI	IR-UR	IR-DI	IR-DR
**5' WT**	171[2]	186	180	181	176	174	171
**ΔY**	167	211	167	165	167	170	176
**ΔZ**	161	157	168	183.	230	170	187
**ΔYZ**	157	151	161	160	218	187	185
**Z-IR**	164	176	148	161	198	179	182

[1] Strains bear the indicated combination of a 3' and 5' fragment of the *cbh1* promoter (compare Tab. 3)

[2] NaOH soluble protein concentration is given in μg/ml.

Table S4 Overview on dry biomass of recombinant strains[1] grown on lactose

	3' WT	ΔX	X-IR	IR-UI	IR-UR	IR-DI	IR-DR
**5' WT**	25.1[2]	14.5	23.5	32.8	23.8	16.9	21.5
**ΔY**	19.1	28.7	28.5	23.6	16.7	18.5	14.7
**ΔZ**	13.4	22.9	22.1	21.4	16.4	17.2	21.3
**ΔYZ**	25.6	23.5	23.1	36.2	23.2	19.3	22.9
**Z-IR**	32.0	31.3	31.0	22.0	23.3	14.9	19.2

[1] Strains bear the indicated combination of a 3' and 5' fragment of the *cbh1* promoter (compare Tab. 3)

[2] Dry weight of biomass is given in mg.

Table S5 Overview on NaOH soluble protein of recombinant strains[1] grown on pre-treated wheat straw

	3' WT	ΔX	X-IR	IR-UI	IR-UR	IR-DI	IR-DR
**5' WT**	333[2]	386	344	339	360	395	359
**ΔY**	384	367	323	404	356	260	444
**ΔZ**	330	333	377	393	360	310	347
**ΔYZ**	369	327	390	416	410	399	353
**Z-IR**	315	319	398	355	336	391	282

[1] Strains bear the indicated combination of a 3' and 5' fragment of the *cbh1* promoter (compare Tab. 3)

[2] NaOH soluble protein concentration is given in μg/ml.

Figure S1 Genotypic characterization of recombinant strains.

(A, B) Numbers indicate the analysed strains according to Table S1. M indicates the GeneRuler 1 kb DNA ladder (Thermo Scientific). (A) Agarose gel electrophoresis of PCRs using chromosomal DNA as template. The first lane of each strain displays the fragment of the 5' flank of the *pyr4* locus obtained with the primer pair 5pyr4_fwd3 and pxyn1_rv_Bam-Nhe (1-3), or the primer pair 5pyr_fwd3 and pcbh1_rv_Bam-Nhe (4-36). Expected fragment sizes can be inferred from Table S1. The second lane of each strain displays the *goxA* fragment obtained with primer pair goxa_fwd_Bam and goxa_rv_Bcu-Nhe (expected size of 1836 bp). The third lane of each strain displays the 3' flank of the *pyr4* locus obtained with primer pair tpyr4_rev2 and pyr4_3fwd (expected size of 1856 bp). (B) Southern blot using *Sac*II digested chromosomal DNA of each strain and goxA as the probe. Numbers, Expected fragment sizes can be inferred from Table S1.

Figure S1

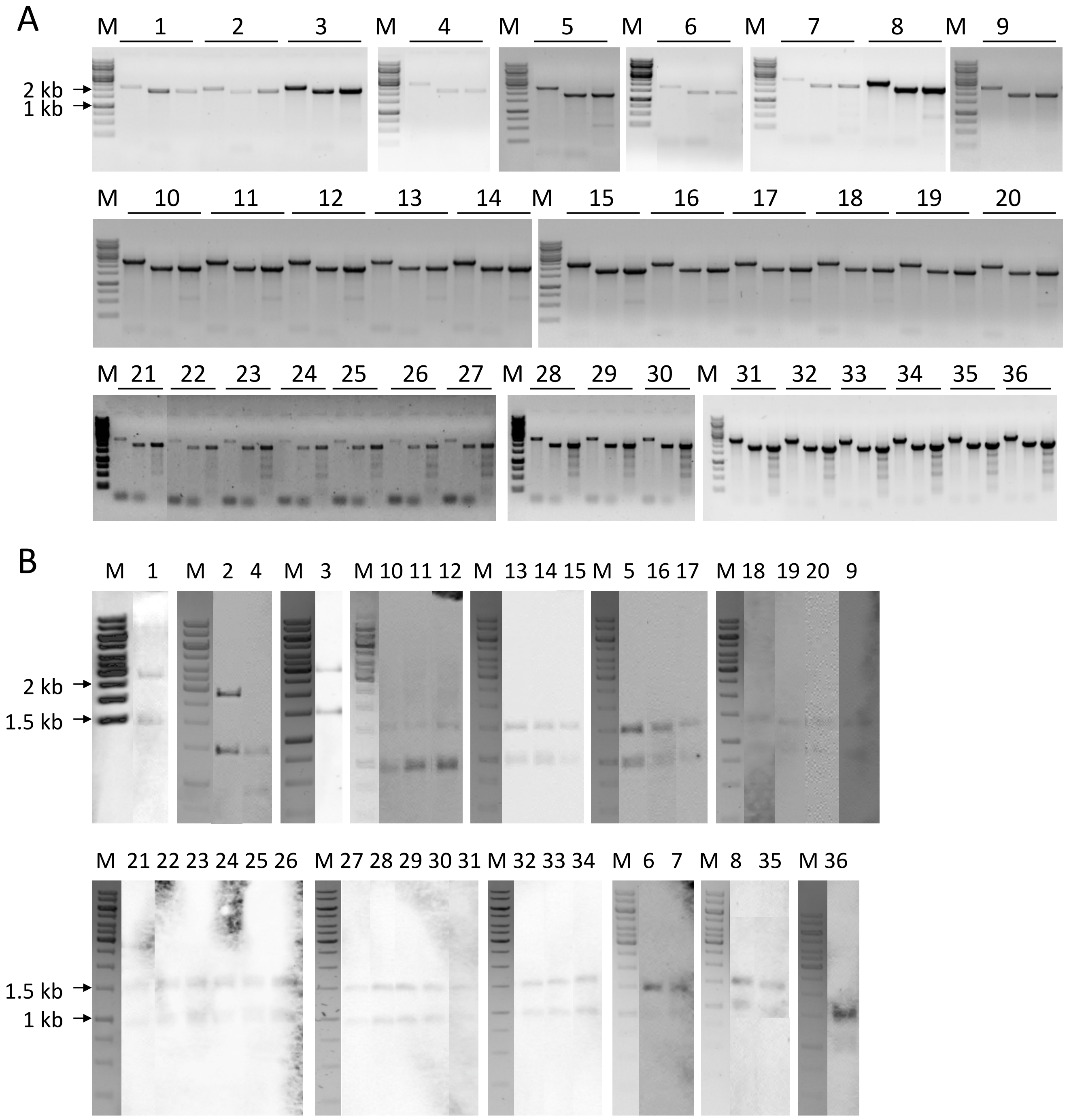
A
M 1 2 3 M 4 M 5 M 6 M 7 8 M 9
2 kb
1 kb
M 10 11 12 13 14 M 15 16 17 18 19 20
M 21 22 23 24 25 26 27 M 28 29 30 M 31 32 33 34 35 36
B
M 1 M 2 4 M 3 M 10 11 12 M 13 14 15 M 5 16 17 M 18 19 20 9
2 kb
1.5 kb
M 21 22 23 24 25 26 M 27 28 29 30 31 M 32 33 34 M 6 7 M 8 35 M 36
1.5 kb
1 kb
